# Integrated small RNA and Degradome sequencing provide insights into salt tolerance in sesame (*Sesamum indicum* L.)

**DOI:** 10.1186/s12864-020-06913-3

**Published:** 2020-07-18

**Authors:** Yujuan Zhang, Huihui Gong, Donghua Li, Rong Zhou, Fengtao Zhao, Xiurong Zhang, Jun You

**Affiliations:** 1 Cotton Research Center, Shandong Academy of Agricultural Sciences, Jinan, 250100 China; 2 Key Laboratory of Biology and Genetic Improvement of Oil Crops, Ministry of Agriculture, Oil Crops Research Institute, Chinese Academy of Agricultural Sciences, Wuhan, 430062 China

**Keywords:** Sesame, Deep sequencing, Degradome miRNA, Salt stress, miRNA–mRNA regulatory network

## Abstract

**Background:**

MicroRNAs (miRNAs) exhibit important regulatory roles in the response to abiotic stresses by post-transcriptionally regulating the target gene expression in plants. However, their functions in sesame response to salt stress are poorly known. To dissect the complex mechanisms underlying salt stress response in sesame, miRNAs and their targets were identified from two contrasting sesame genotypes by a combined analysis of small RNAs and degradome sequencing.

**Results:**

A total of 351 previously known and 91 novel miRNAs were identified from 18 sesame libraries. Comparison of miRNA expressions between salt-treated and control groups revealed that 116 miRNAs were involved in salt stress response. Using degradome sequencing, potential target genes for some miRNAs were also identified. The combined analysis of all the differentially expressed miRNAs and their targets identified miRNA–mRNA regulatory networks and 21 miRNA–mRNA interaction pairs that exhibited contrasting expressions in sesame under salt stress.

**Conclusions:**

This comprehensive integrated analysis may provide new insights into the genetic regulation mechanism of miRNAs underlying the adaptation of sesame to salt stress.

## Background

Sesame (*Sesamum indicum* L.) is an important high-quality oil crop in the world, and is grown in more than 70 countries mainly in Asia and Africa, including India, Myanmar, China, Sudan and Ethiopia (FAO, 2018). The world’s annual planting area is about 10.7 million hectares, with a total output of more than 6 million tons (FAO, 2018). Sesame seeds are rich in oil with high levels of unsaturated fatty acids (oleic and linoleic), protein, and especially high levels of methionine and micronutrients, including lignans, minerals, phytosterol and tocopherol [1, 2]. The nutritional, medicinal and industrial values of sesame seeds have been recognized by increasing numbers of people in recent years [3, 4]. Compared to other oil crops, sesame is moderately tolerant to salinity stress, but salt-tolerant (ST) varieties are urgently needed in salt-affected zones like inland and coastal saline areas of major sesame production countries [5]. Therefore, it is necessary to understand the molecular mechanisms involved in the salt stress response in sesame, to aid the development of basic research and selection of ST varieties.

It has been well established over many years that microRNAs (miRNAs) can regulate gene expression by cleavage or translational repression of the mRNA of target genes [6]. In plants, miRNAs exhibit diverse and important roles in plant growth, development and metabolism, as well as responses to various abiotic stresses, such as salinity, drought, and cold, among others [7, 8] . Increasing numbers of recent studies have suggested the importance of miRNAs in plant response to salt stress, including for *Arabidopsis* [9], cotton [10], eggplant [11], maize [12], radish [13], rice [14], sugarcane [15], *Thellungiella salsuginea* [16] and wheat [17]. Furthermore, many miRNAs and their target genes have been identified by small RNA and degradome deep sequencing, suggesting that high-throughput sequencing should provide a clear opportunity to identify and validate miRNA–mRNA pairs in plants [18–20]. For instance, one report found that five miRNAs were involved in salt stress response in *Hordeum bulbosum* using deep sequencing, and demonstrated their critical roles in better tolerance to salt stress in autopolyploids [21] . It was also clearly demonstrated that overexpression of some miRNAs or their target genes in some transgenic plants resulted in enhanced resistance to salt stress, such as for gma-miR172c [9], miR319 [22], miR393 [23], miR394 [24], miR395e [25], Sp-miR396a-5p [26], ghr-miR414c [27] and osa-miR528 [28]. Currently, all of the evidence indicates the importance of miRNA regulation in plant salt tolerance; however, the function of salt-responsive miRNAs and their target genes in sesame remain unknown.

In the present research, to investigate the response of sesame to salt stress at the mRNA and miRNA levels, small RNA and degradome deep sequencing were performed on seedlings of ST and salt-sensitive (SS) sesame genotypes during the early stage (12 and 24 h) of salt stress. These two contrasting genotypes were used to help us understand the salinity response and tolerance mechanisms of sesame by analyzing miRNA expression patterns and their target genes.

## Results

### Small RNA sequencing and annotation of sesame miRNAs

**Fig. 1:**
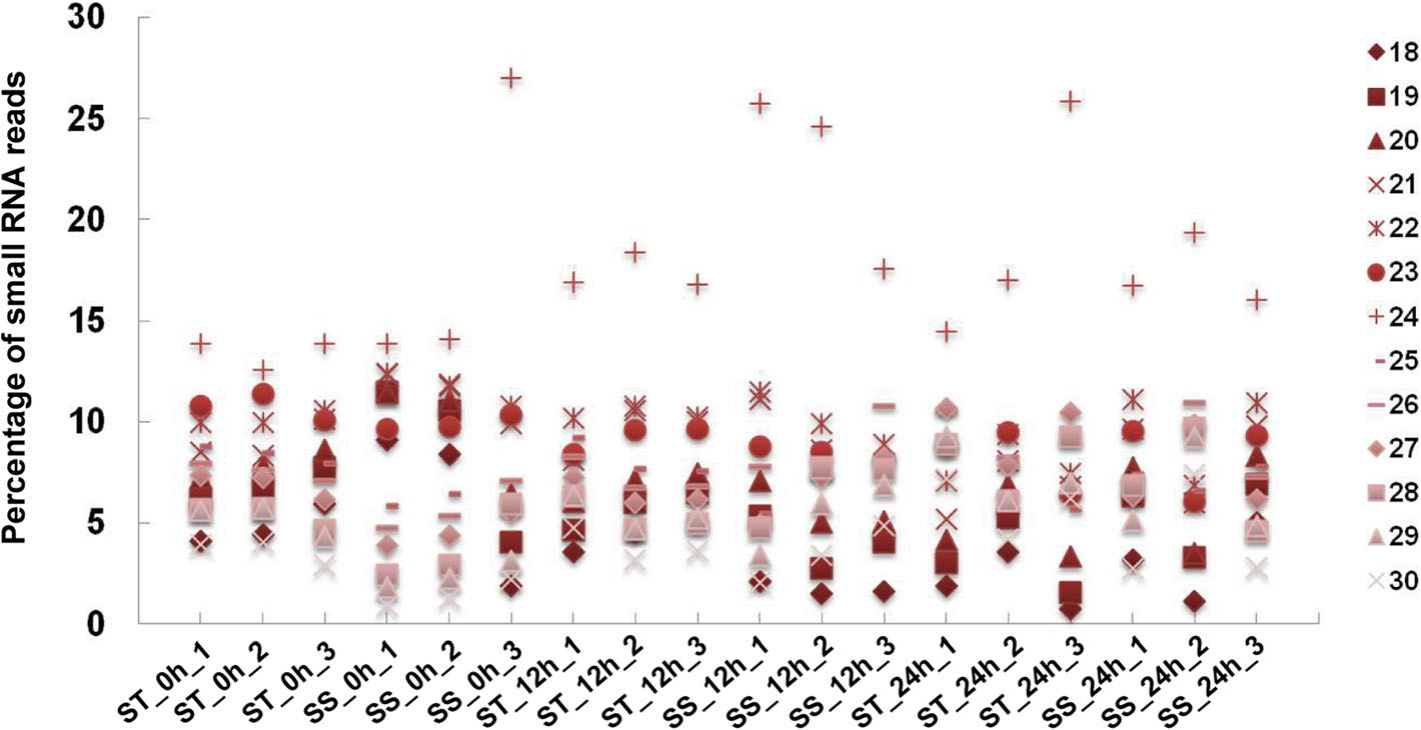
Length distribution of small RNAs (18–30 nt) from the 18 libraries in sesame

In the study, 18 small RNA libraries of ST and SS genotypes under control and salt stress conditions (12 and 24 h) were constructed and sequenced. A statistical summary of sequencing results for the 18 sRNA libraries is shown in Table S1. A total of 229.4 million raw reads with an average of 12.7 illion reads for each sample were generated (Table S1). After quality control and length selection of the reads, a set of 222.6 million clean reads ranging within 18–30 nt was obtained from these samples for further analysis (Table S1). The length distribution of characterized sequences from these libraries is shown in Fig. 1, and the most abundant class was reads of 24 nt.

After the filtered reads were searched against the miRNAs of other plant species from miRBase22, a total of 351 conserved/known miRNAs were identified in sesame. These miRNAs were clustered into 64 families according to sequence similarity (Table S2). Among these families, miR156 was the largest family with 30 members, followed by miR396 with 26 members. Further, based on the method for novel miRNA prediction, a final set of 94 unique novel miRNAs with their hairpin precursors was obtained (Table S2). Detailed information of miRNAs and their precursor sequences are listed in Table S2. In general, the average GC content of mature miRNA sequences was about 49.4% and their length ranged within 18–25 nt, with 20 and 21 nt being most abundant.

### Differential expression of miRNAs under salt stress

**Fig. 2:**
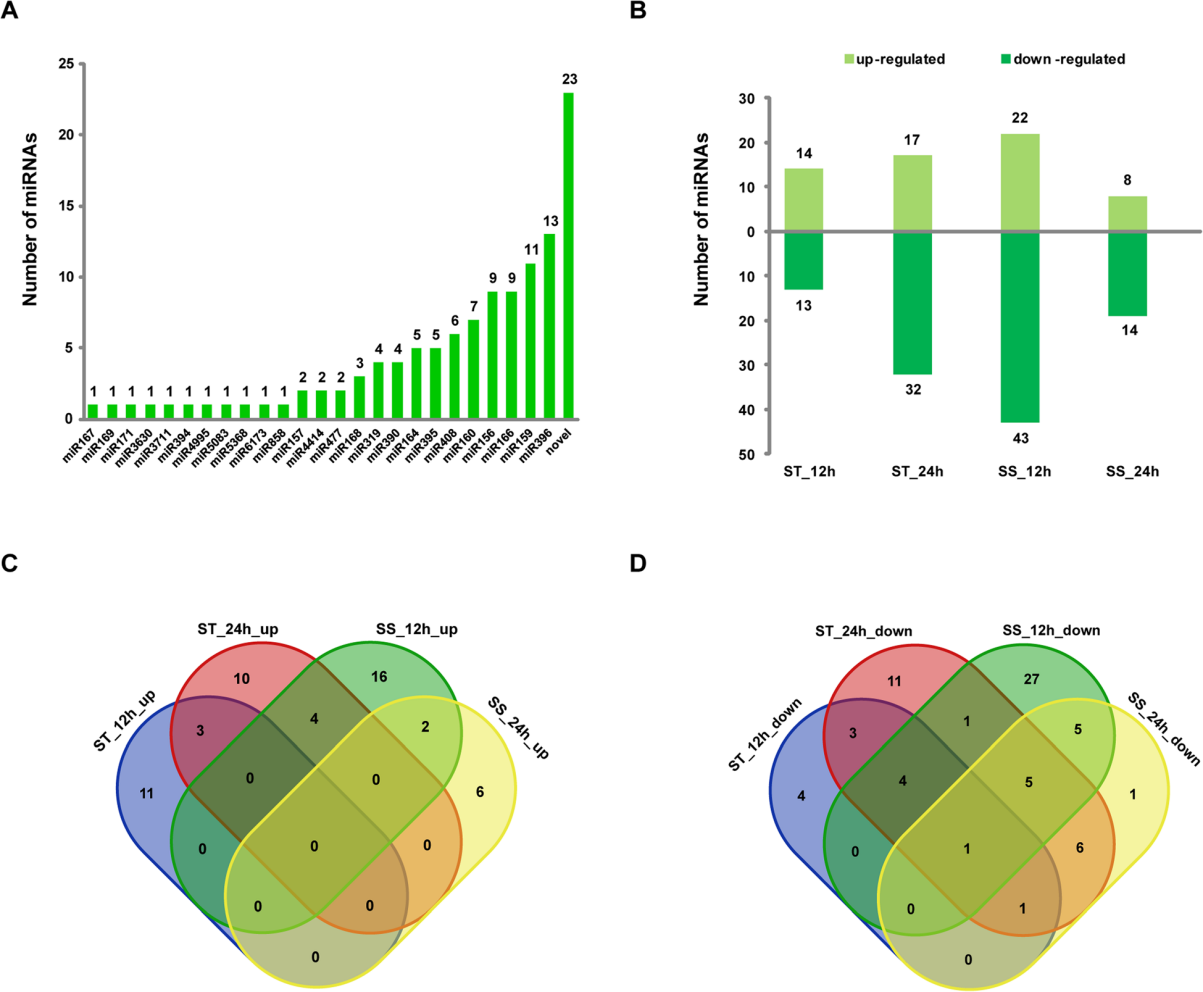
The number of differentially expressed miRNAs in ST and SS genotype responses to salt stress. **(A)** The number of miRNA family members. **(B)** Numbers of differentially expressed miRNAs in ST and SS at different salt stress time points. Venn diagrams of up- **(C)** and down-regulated miRNAs **(D)** in ST and SS under salt stress

**Fig. 3:**
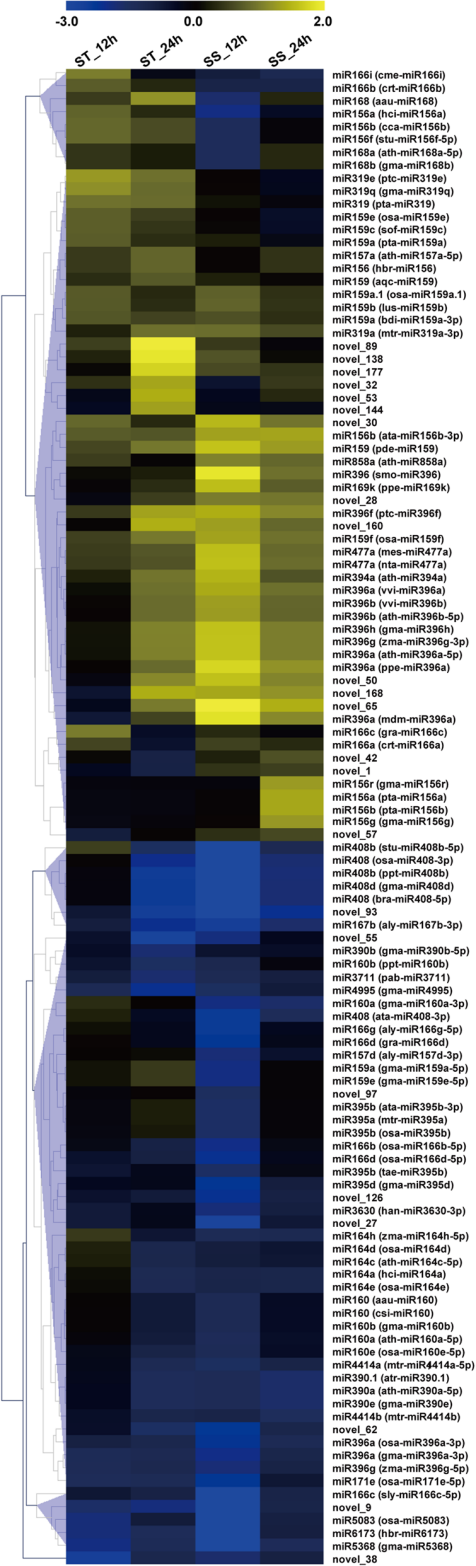
Expression profiles of differentially expressed miRNAs in ST and SS genotypes under salt stress. Data are presented as heatmap of a log_2_ transformed fold change

In this study, a total of 116 differentially expressed miRNAs were identified in the two sesame accessions under salt stress. Among these miRNAs, 93 known miRNAs belonging to 25 families and 23 novel miRNAs were differentially expressed upon salt stress (Fig. 2A). The miR396 was clearly one of the largest miRNA families with 13 members in sesame response to salt stress, followed by miR159, miR166 and miR156 families (Fig. 2A). Compared with control (0 h), 14 and 17 miRNAs were identified as significantly up-regulated in ST at 12 and 24 h, respectively, and the corresponding values for SS were 22 and 8 (Fig. 2B). Meanwhile, a set of 13 and 32 miRNAs were down-regulated in ST at 12 and 24 h, respectively, and correspondingly for SS there were 43 and 14. Thus, the majority of miRNAs were down-regulated in the sesame response to salt stress (Fig. 2B). Overall, many differentially expressed miRNAs exhibited genotype- and time-specific expression at the different sampling times under stress (Fig. 2C and D). Further, hierarchical clustering analysis highlighted several distinctive expression patterns of salt-responsive miRNAs (Fig. 3). For example, a total of 13 conserved miRNAs (miR156, miR156b/cca-miR156b, miR156f, miR157a, miR159/aqc-miR159, miR159a/pta-miR159a, miR159e/osa-miR159e, miR166b/crt-miR166b, miR166c/gra-miR166c, miR166i, miR319, miR319e and miR319q) and six novel miRNAs (novel_32, novel_53, novel_89, novel_138, novel_144 and novel_177) were up-regulated only in ST genotype under salt stress (Fig. 3).

To further validate the reliability of the data produced through small RNA sequencing, the relative expression levels of 10 differentially expressed miRNAs randomly selected from the expression profile data were examined using quantitative real-time PCR (qRT-PCR)– showing close agreement between the two results (r^2^ = 0.80; Fig. S1).

### Identification of miRNA targets by degradome analysis

**Fig. 4:**
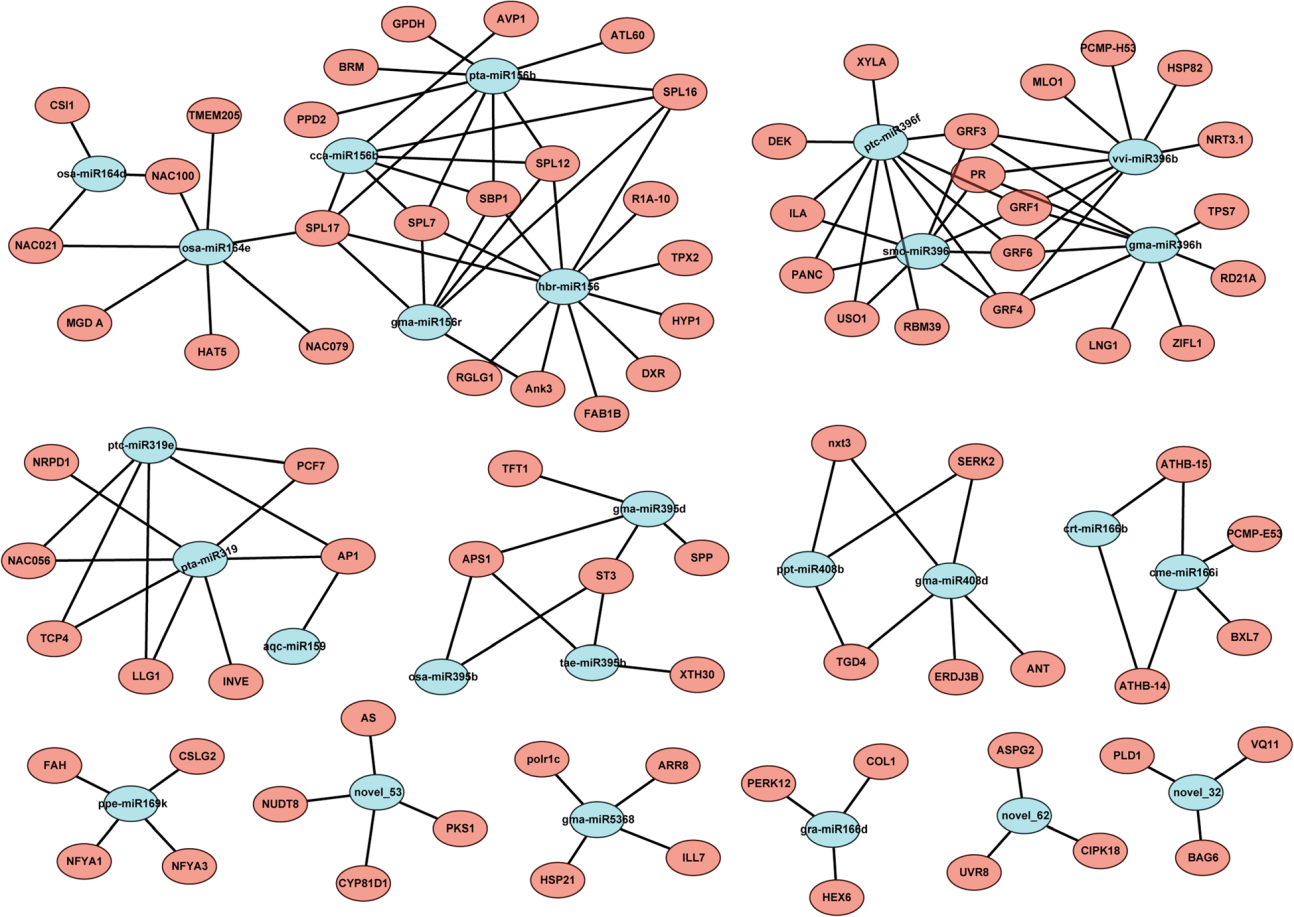
MiRNA-mediated regulatory networks in ST and SS genotype responses to salt stress. Blue circles represent the differentially expressed miRNAs and red circles represent the target genes of differentially expressed miRNAs. Abbreviations for target genes are listed in Table S5

Plant miRNAs are known to have perfect or near-perfect complementarity with their targets and can be predicted using both computational tools and sequencing approaches like degradome analysis. To better investigate the regulatory functions of miRNAs, their target genes were identified using degradome sequencing, and the summary of sequencing data is shown in Table S3. Through degradome analysis, a total of 718 targets were identified for 302 miRNAs (containing 283 known and 19 novel miRNAs) in the present study (Table S4). Generally, many miRNAs targeted multiple genes and most were highly conserved. For instance, many squamosa promoter binding protein-like genes observed in the study were targeted by both miR156 and miR157 families, and miR164 members targeted genes encoding some NAC and GTE7-like transcription factors (Table S4). Degradome cleavage site and alignment range of target genes are also listed in Table S4. In addition, degradome analysis showed that 210 genes were the targets of 85 differentially expressed miRNAs in sesame (Table S5). Through expression analysis of target genes in response to salt stress in sesame, a large number of members of the NAC, MYB, TCP and auxin response factor (ARF) families were ranked top in the context of salinity response, indicating their important roles for sesame in combating salt stress (Table S5). Further, network analysis of salt-responsive miRNAs and their target genes showed that some important miRNA–target gene regulatory models included several miRNA family members and some stress-related genes encoding ATP sulfurylase 1 (APS1), growth-regulating factors (GRFs), NAC transcription factors, homeobox-leucine zipper proteins (ATHBs), low affinity sulfate transporter 3 (ST3) and transcription factor TCP 4 (Fig. 4).

### Functional enrichment analysis of miRNA target genes

**Fig. 5:**
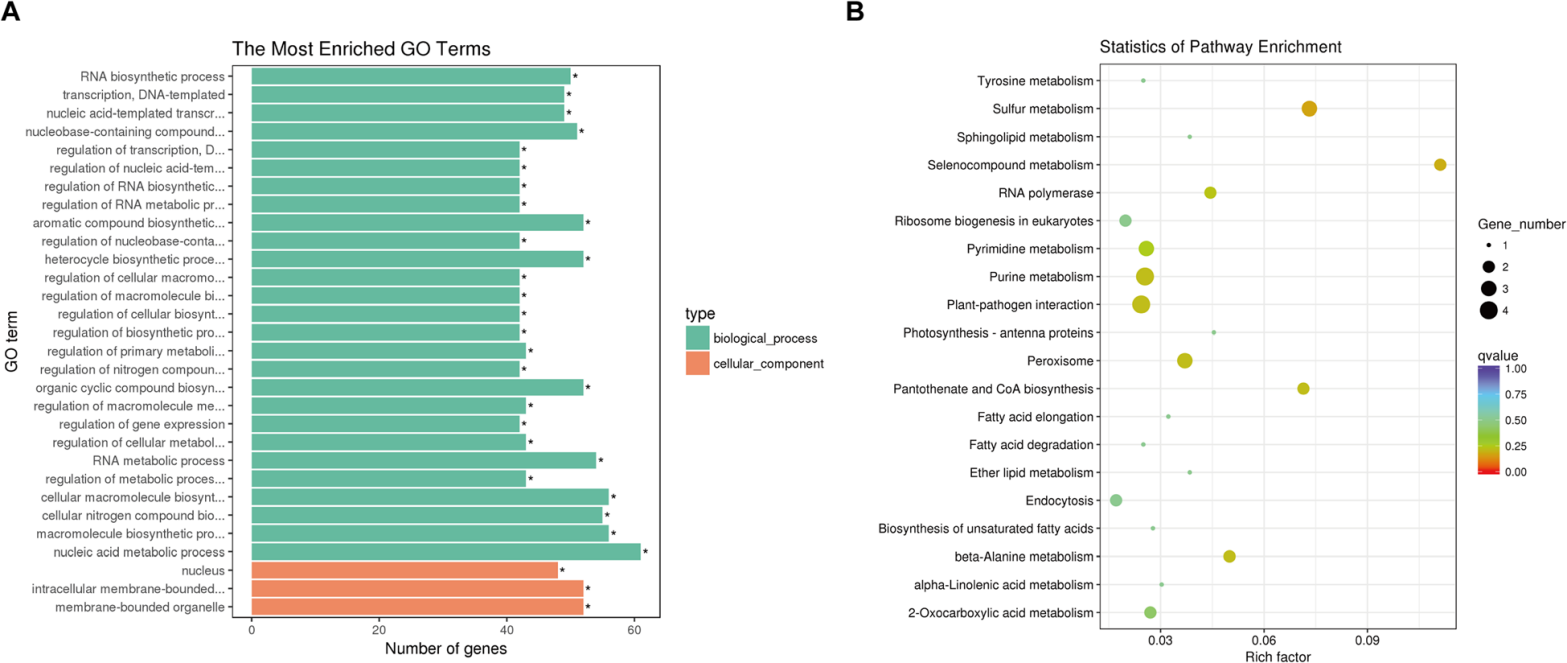
GO classification (A) of miRNA targets and top of 20 enriched KEGG pathways (B)

In order to investigate the regulatory functions of miRNAs in response to salt stress in sesame, a total of 216 salt-responsive miRNA targets were analyzed with Gene Ontology (GO) functional classification and Kyoto encyclopedia of genes and genomes (KEGG) pathway enrichment (Fig. 5, Table S4). First, GO enrichment analysis showed that the most enriched biological processes were aromatic compound biosynthetic process (GO:0019438), heterocycle biosynthetic process (GO:0018130), regulation of biosynthetic process (GO:0009889), regulation of metabolic process (GO:0019222) and nucleic acid metabolic process (GO:0090304) (Fig. 5A). Among cellular components, the most significant GO terms were involved in nucleus (GO:0005634), intracellular membrane-bounded organelle (GO:0043231) and membrane-bounded organelle (GO:0043227) (Fig. 5A). Second, based on the KEGG analysis, the most enriched pathways in response to salt stress were those related to sulfur metabolism (sind00920), selenocompound metabolism (sind00450), pantothenate and CoA biosynthesis (sind00770), peroxisome (sind04146), purine metabolism (sind00230) and plant–pathogen interaction (sind04626) (Fig. 5B).

### Correlation analysis of salt-responsive miRNAs and target expression profiles

**Fig. 6:**
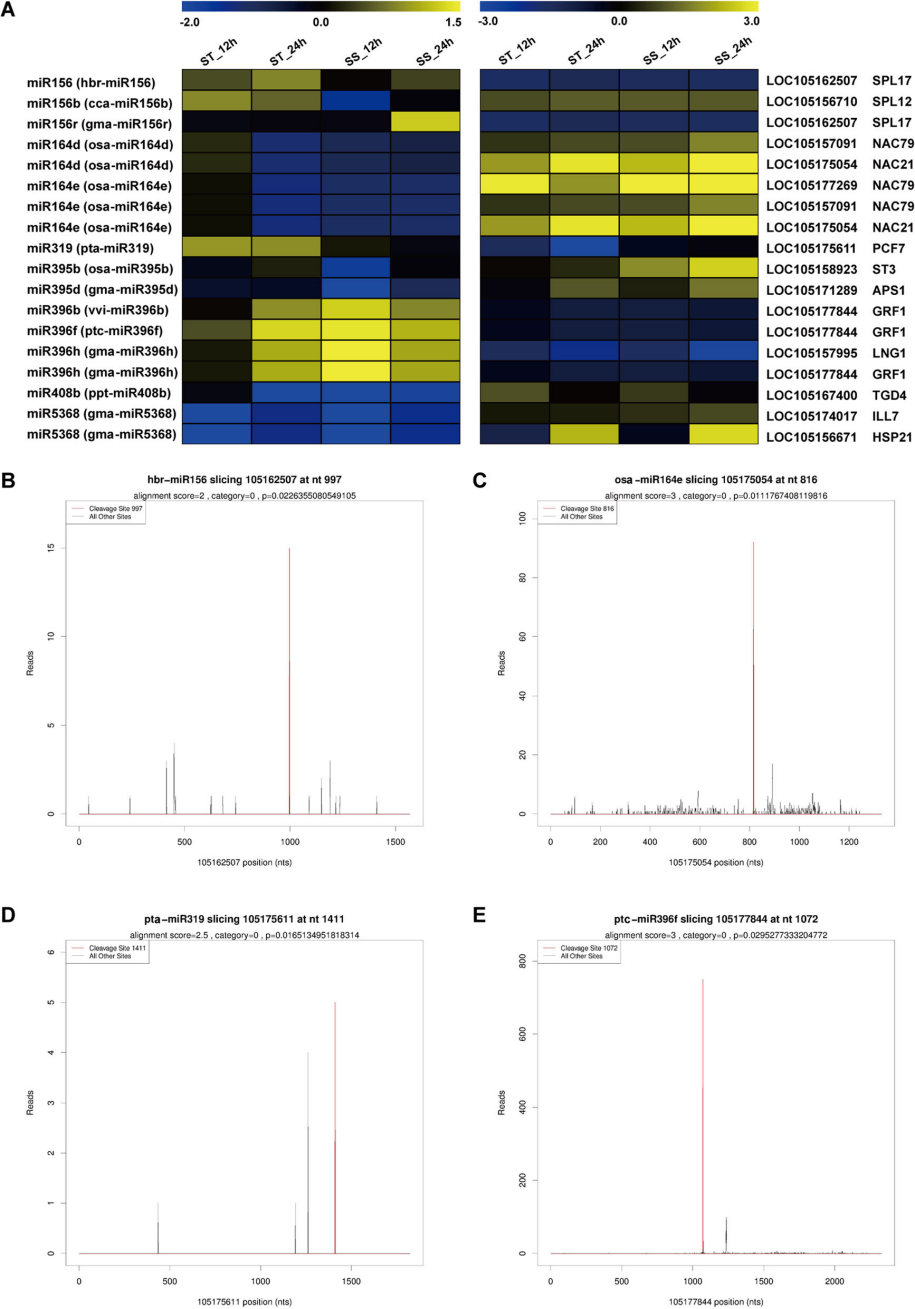
Expression profiles (A) of important miRNA–mRNA interaction pairs in ST and SS genotypes and examples of t-plots (B–E) of some miRNA targets confirmed by degradome sequencing. Data are presented as heatmaps of a log_2_ transformed fold change. The left side of the heat map represents the relative expression of miRNAs, and the right side shows the relative expression levels of corresponding target genes in ST and SS genotypes under salt conditions at two time points. Abbreviations for target genes are listed in Table S5

**Fig. 7:**
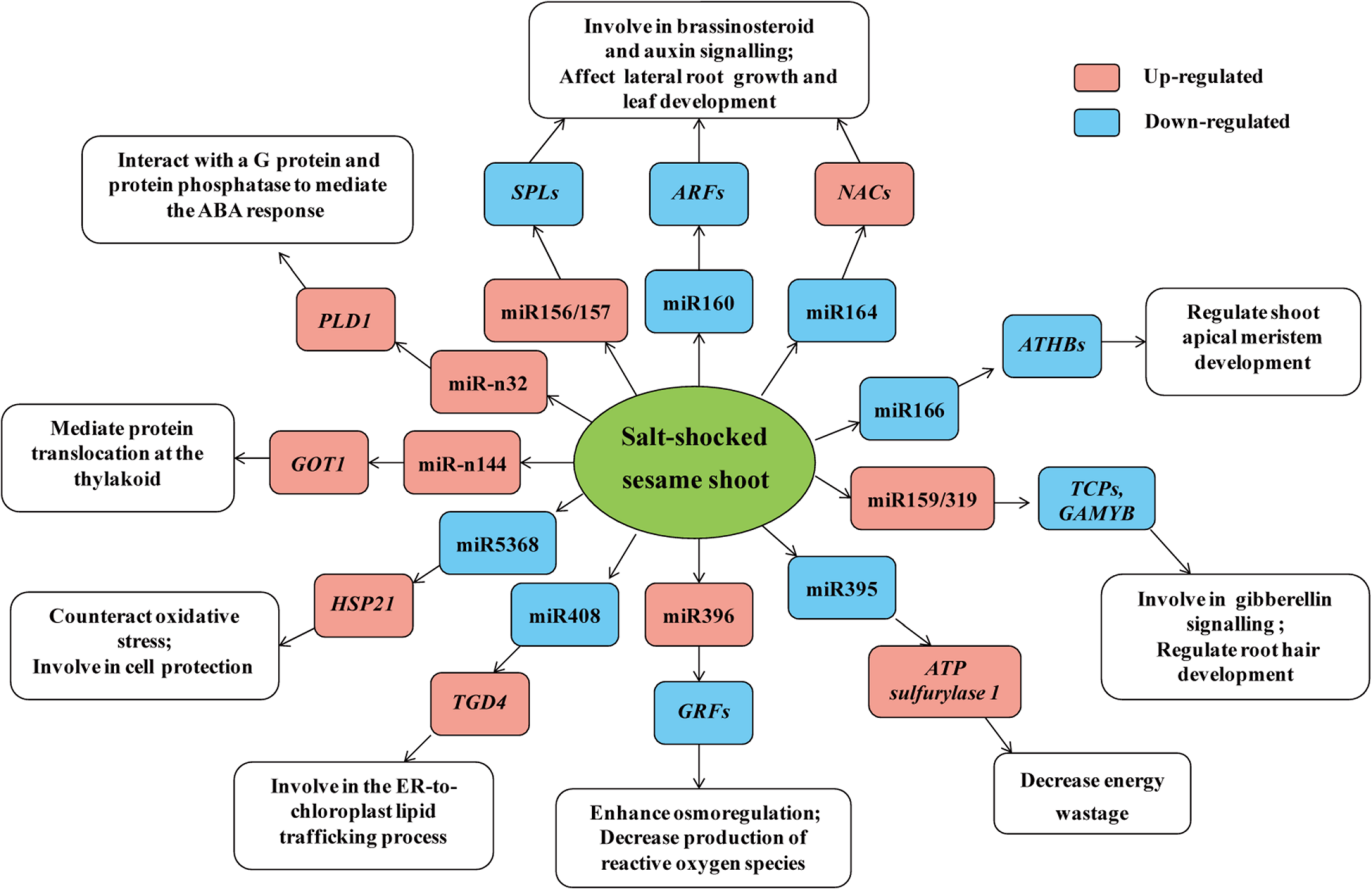
Model showing the responses and tolerance of sesame based on miRNA–mRNA regulatory network. Abbreviations for target genes are listed in Table S5

The accumulation of miRNA, in general, leads to down-regulation of their target genes and vice-versa. The expression patterns of miRNA target genes in response of ST and SS genotypes to salt stress were also analyzed, and details are listed in Table S5. To further investigate the association of salt-responsive miRNAs with their targets, correlation analysis of their expression profiles was performed. A total of 1050 miRNA–mRNA interaction pairs were identified across all comparisons between the control (0 h) and treatment groups (12 and 24 h) in ST and SS genotypes. Out of these pairs, 579 were negatively correlated, however, only 21 miRNA–mRNA pairs were significantly negatively correlated (*P* < 0.05) (Table S6). Negative correlations were those in which miRNA expression was higher when expression of target mRNA was lower and vice-versa. In contrast, positive correlations indicated that they had similar expressions. Furthermore, among the negatively correlated pairs, 18 important miRNA–mRNA pairs exhibited contrasting expression changes at 12 or 24 h in ST or SS genotype responses to salt stress (Fig. 6A). The miRNA–mRNA pairs in this study were also validated by degradome sequencing. Figure 6B–E shows several examples of t-plots of miRNA targets. A model of the responses to salt stress of the two sesame genotypes based on the miRNA–mRNA regulatory network shows the potential roles of the salt-responsive miRNAs in the early phase of salt stress (Fig. 7).

## Discussion

Soil salinization is one of the main problems in global agricultural production, which seriously affects the growth and development of agricultural crops [29]. Salt tolerance of plants is a complex quantitative trait and is controlled by multiple genes, involving a variety of physiological and biochemical processes, and is coordinated by a variety of salt-tolerance mechanisms, in which miRNAs are involved [7, 30]. In sesame, just a few miRNAs were identified [31], there is no study reporting the genome-wide discovery of miRNAs and characterizing the role of miRNAs in response to salt stress. Here, to better understand the genetic and molecular mechanisms underlying salt stress response in sesame, we characterized both miRNAs and their target genes in ST and SS genotypes using small RNA and degradome sequencing and analyzed their expression profiles during the early stage of salt stress. A total of 116 miRNAs and 210 target genes were found involved in the salt stress response – their molecular functions, regulatory networks and main mediated pathways are further discussed below.

### Salt-responsive miRNAs in sesame

A large number of salt-responsive miRNAs and their targets have been identified from plant species, including many conserved miRNAs such as miR171, miR172, miR319, miR393 and miR396 families [7, 9, 22, 26, 32]. Our study confirmed most previously known salinity-responsive miRNAs. Previous studies in other plant species have shown genotype-specific miRNA expression profiles in response to salt stress [12, 33]. In sesame, three conserved miRNAs (miR319, miR319e and miR319q) showed up-regulation in ST genotype in the 12 and 24 h samples, but miR159 (pde-miR159) in the SS genotype was up-regulated during salt treatment (Fig. 3). Furthermore, we also identified some novel miRNAs in sesame related to salt stress response. A total of 23 novel miRNAs were induced strongly in at least one sample after salt stress, and their expression levels changed significantly in ST or SS genotypes under salt stress. In particularly, six novel miRNAs (novel_32, novel_53, novel_89, novel_138, novel_144 and novel_177) were significantly up-regulated in ST genotype under salt stress, but novel_1 and novel_42 showed down-regulation. It is suggested that these specific-expression conserved and novel miRNAs may be involved in the regulation network of sesame response to salinity stress and play critical roles in regulating gene expression and metabolism processes during salinity treatment. Although a large number of salt-responsive miRNAs are conserved in plants to a certain extent, some miRNAs have different regulatory patterns among different species. For example, after salt stress treatment, miR396 was significantly up-regulated in *Arabidopsis* [34], but down-regulated in maize [12] and cotton [32]. In this study, we also found that three miR396 members showed down-regulation in sesame under salt stress, but another 10 members were up-regulated. Among different plant species, the same miRNA has different expression patterns, indicating that species-specific miRNA regulation mechanism may have formed in the process of long-term evolution, and it is necessary to analyze the mechanism of miRNA involvement in salt stress response in various plants.

### MiRNA–mRNA regulatory network in sesame under salt stress

In this study, we found that salt-responsive miRNA target genes were involved in DNA binding, transcriptional regulation and other processes, suggesting that miRNA plays an important role in the regulation of transcriptional processes in salt stress responses. Most current research has found that miRNA may not only regulate multiple target genes, but also a single gene can be jointly regulated by multiple miRNAs – this relationship is called the miRNA–target gene regulatory model [35–37]. The diversity and complexity of miRNA regulation indicates their importance in biological processes, and many miRNA regulation modules could constitute a complex miRNA–mRNA regulatory network. Therefore, research on miRNA–mRNA regulatory networks could provide useful information for understanding complex biological processes, which is of great significance to further study of plant salt-tolerance mechanisms. In this study, a number of novel and conserved miRNAs were involved in the regulatory network in sesame adaptive response to salt stress by regulating specific stress-related genes, such as novel_32, novel_53, novel_62 and some family members of miR156, miR159, miR164, miR166, miR319, miR395, miR396 and miR408 (Fig. 4). Among them, several examples are miR156-mediated cleavage of SPLs, miR164-mediated cleavage of NACs, miR319-mediated cleavage of TCPs and miR396-mediated cleavage of GRF genes. Previous studies have shown that NAC family members (NAC1, NAC4, NAC13 and NAC17) play important regulatory roles in plant responses to salt stress via activating the expression of stress-responsive genes [38–41]. In our present work, we found that the level of NAC 21 and NAC79 mRNA increased compared with the decrease in miR164d and miR164e in sesame under salt stress, suggesting their important roles in sesame (Fig. 6). Another study also showed that Sp-miR396a-5p transcript level in Solanaceae was up-regulated under salt stress, and overexpression of Sp-miR396a-5p in tobacco increased its tolerance to salt stress by enhancing osmoregulation and decreased production of reactive oxygen species [27]. In this study, we observed that expression of miR396b, miR396f and miR396h increased in sesame with the decrease of their target gene *GRF1* at 12 and 24 h after salt stress, indicating that miR396 might have a similar regulatory function in sesame resistance to salt stress. Generally, miRNA may be the center of a complex network of gene regulation, and their target genes in this miRNA–mRNA regulatory network might play critical roles in the regulation of sesame responses to salt stress.

### MiRNA-mediated regulatory/signaling pathways involved in response to salt stress

In plants, the adverse effects of salt stress are mainly hyperosmotic stress, sodium ion toxicity and oxidative stress [42–44]. To tolerate salt stress, plants have developed a series of adjusting methods, including sodium ion isolation and exclusion, abscisic acid-mediated stomatal movement, accumulation of specific osmoregulation substances and scavenging systems for reactive oxygen species [45–48]. Similarly, through GO and KEGG enrichment analysis, many salt-responsive miRNA target genes in sesame were found to be involved in regulation of biosynthetic process, metabolic process, cellular macromolecule biosynthetic process, peroxisome, α-linolenic acid metabolism, amino acid biosynthesis and metabolism, carbohydrate and energy metabolism, and other important biological and metabolic pathways (Fig. 5). In addition, almost all plant hormones and their signaling pathways play important roles in response of plants to abiotic stresses [49]. In this study, several miRNAs, including family members of miR156, miR160 and miR319 were found to participate in brassinosteroid, auxin and gibberellin mediated-signaling pathways by regulating their target genes, respectively (Table S4). Especially, seven ARF genes (including *ARF17* and *ARF18*) were identified to be targeted by several members of the miR160 family based on degradome sequencing, and most were significantly down-regulated in salt treatment relative to the control (Table S5). Similar findings of miR160-mediated ARF regulation were also reported in other plant responses to salt, drought and cold stresses [50–52]. These results suggest that these salt-responsive miRNAs may act as pivotal factors within the whole process of sesame responses to salt stress, mediating the regulation of multiple gene expressions and thus participating in various regulatory/signaling pathways. Thus, the regulation mechanisms of most miRNAs remain unclear, and more functional studies are needed to elucidate the roles of miRNAs in response to salt stress using transgenic technology.

## Conclusions

The present study is the first attempt to integrate miRNA and mRNA expression data along with degradome analysis to identify key regulatory miRNA–mRNA circuits in sesame in response to salt stress. A total of 445 miRNAs (351 known and 94 novel) with precursors were identified in sesame using deep sequencing. Of these miRNAs, 116 were expressed differentially in ST and SS genotypes after salt stress. In addition, the potential miRNA target genes were verified by degradome sequencing, and 21 miRNA–mRNA pairs showed contrasting expression patterns in sesame response to salt stress. Overall, the comprehensive integrated analysis in this study not only provides important information for transcriptome dynamics and regulatory network components of salinity response and adaptation in sesame, but is also an important reference for genetic improvement of salt tolerance in sesame and other plants.

## Methods

### Plant materials and RNA extraction

In this study, two sesame accessions WZM3063 (ST) and ZZM4028 (SS) were provided by the China National Genebank, Oil Crops Research Institute, Chinese Academy of Agricultural Sciences. The growth of sesame seedlings and the salt treatment were performed as previously described [53]. The sesame shoot samples with three biological replicates were collected at 0 (control), 12 and 24 h of salt stress. All samples were stored at − 80 °C until total RNA isolation. Total RNA of 18 samples was extracted according to a previously reported method [53] and used for small RNA, degradome sequencing and quantitative real-time PCR (qRT-PCR) assay.

### Small RNA library construction, sequencing and data analysis

Small RNA libraries were constructed and sequenced by a high-throughput sequencing method (on an Illumina HiSeq 2500 platform) at Novogene (Beijing, China), according to previously published methods [53]. Quality control and analysis of sequencing data were also performed by Novogene using methods described previously [54, 55]. The miRBase (version 22) was used as a reference to search for known miRNAs by software miRDeep2, and srna-tools-cli was used to obtain the potential miRNAs and draw the secondary structures [56]. Only perfect matches were considered known miRNAs and these miRNAs are grouped into families based on the similarity of the mature miRNA sequences using miFam.dat (http://www.mirbase.org/ftp.shtml). Reads that were not aligned to the miRBase database were used to predict novel miRNAs. Potential novel miRNA prediction was performed based on their precursors and the hairpin RNA structures by using miREvo [57] and miRDeep2 [56]. For each miRNA, the abundance in different libraries was calculated and normalized to transcripts per million using global normalization procedures [58]. Differential expression analysis of the two groups was performed using the DESeq R package (1.8.3) with *P* < 0.05 considered significant [59].

### Degradome library sequencing and target identification

For degradome sequencing, equal amounts of total RNA from each ST sample were mixed to build one library, while equal masses of total RNA from different SS samples were mixed as another library. Two degradome libraries were constructed according to the manufacturer’s instructions and then sequenced on an Illumina HiSeq 2500 platform at LC-BIO (Hangzhou, China). Subsequent data analysis, including data processing, prediction of miRNA cleavage sites and target plot (t-plots) figure drawings, was based on previous reports [60, 61]. The sesame genome v.1.0 (https://www.ncbi.nlm.nih.gov/genome/?term=sesamum) was used as a reference in this study. The miRNA–mRNA network construction was presented using Cytoscape 3.7.2.

### Expression and function analysis of the potential miRNA targets

Expression analysis of all the target genes was performed using transcriptomic data of sesame in response to salt stress described in detail in our previous report [53]. GO enrichment analysis of all the identified targets of differentially expressed miRNAs was performed using GOseq [62] to uncover the miRNA–gene regulatory network. The KEGG pathway for the target genes was analyzed through KOBAS (2.0) software. Finally, correlation analysis of miRNA expression profiles and their target genes was performed using the software R version 3.1.1.

### qRT-PCR

To validate the reliability of the high-throughput sequencing data, qRT-PCR of 10 selected miRNAs was performed. Reverse transcription and qRT-PCR reactions were performed using a miRcute Plus miRNA First-Strand cDNA Kit (Tiangen Biotech, Beijing, China) and a miRcute Plus miRNA qPCR Kit (SYBR Green, Tiangen Biotech) according to the manufacturer’s instructions, respectively. The U6 snRNA was used as the reference gene for normalizing miRNA expression. Sequences of primers used for qRT-PCR in this study are presented in Table S7. The relative expression levels of miRNAs were calculated using the 2^−∆∆Ct^ method [63]. Three technical replicates were performed for each reaction. Correlation analysis of miRNA expression profiles between high-throughput sequencing and qRT-PCR was performed using R version 3.1.1.

## Supplementary information

**Additional file 1: Table S1.** Summary of small RNA sequencing data generated from 18 small RNA libraries. **Table S2.** Details of identified miRNAs (known and novel) and their precursors. **Table S3.** Summary of degradome sequencing data. **Table S4.** List of miRNA targets identified in sesame by degradome sequencing. **Table S5.** The expression patterns of miRNAs and their target genes in ST and SS genotype responses to salt stress. **Table S6.** Correlation analysis between the expression profiles of miRNAs and their target genes. **Table S7.** Primers used in this study.

**Additional file 2 Fig. S1.** Correlation analysis between qRT-PCR and small RNA sequencing data based on log_2_fold change of 10 selected miRNAs.

## Data Availability

The raw sequence data of small RNA and degradome libraries were respectively deposited in NCBI Sequence Read Archive (SRA, www.ncbi.nlm.nih.gov/sra) with accession numbers PRJNA629518 and PRJNA629631.

## Abbreviations

APS1: ATP sulfurylase 1
ATHBs: Homeobox-leucine zipper proteins
*FDR*: False Discovery Rate
*FPKM*: Fragment Per kb per Million reads
GO: Gene Ontology
GRFs: Growth-regulating factors
*KEGG*: Kyoto encyclopedia of genes and genomes
*miRNA*: MicroRNA
qRT-PCR: Quantitative real-time PCR
SS: Salt-sensitive
ST: Salt-tolerant
ST3: Sulfate transporter 3
T-plot: Target plot

## References

[1] Namiki M (2007). Nutraceutical functions of sesame: a review. Crit Rev Food Sci Nutr.

[2] Majdalawieh AF, Massri M, Nasrallah GK (2017). A comprehensive review on the anti-cancer properties and mechanisms of action of sesamin, a lignan in sesame seeds (*Sesamum indicum*). Eur J Pharmacol.

[3] Zhou L, Lin X, Abbasi AM, Zheng B (2016). Phytochemical contents and antioxidant and Antiproliferative activities of selected black and white sesame seeds. Biomed Res Int.

[4] Majdalawieh AF, Mansour ZR (2019). Sesamol, a major lignan in sesame seeds (*Sesamum indicum*): anti-cancer properties and mechanisms of action. Eur J Pharmacol.

[5] Li D, Dossa K, Zhang Y, Wei X, Wang L, Zhang Y, Liu A, Zhou R, Zhang X: GWAS Uncovers Differential Genetic Bases for Drought and Salt Tolerances in Sesame at the Germination Stage. Genes 2018, 9(2). 10.3390/genes9020087 PMC5852583 29443881

[6] Van Ex F, Jacob Y, Martienssen RA (2011). Multiple roles for small RNAs during plant reproduction. Curr Opin Plant Biol.

[7] Kumar R (2014). Role of microRNAs in biotic and abiotic stress responses in crop plants. Appl Biochem Biotechnol.

[8] Borges F, Martienssen RA (2015). The expanding world of small RNAs in plants. Nat Rev Mol Cell Biol.

[9] Li W, Wang T, Zhang Y, Li Y (2017). Overexpression of soybean miR172c confers tolerance to water deficit and salt stress, but increases ABA sensitivity in transgenic *Arabidopsis thaliana*. J Exp Bot.

[10] Gao S, Yang L, Zeng HQ, Zhou ZS, Yang ZM, Li H, Sun D, Xie F, Zhang B (2016). A cotton miRNA is involved in regulation of plant response to salt stress. Sci Rep.

[11] Zhuang Y, Zhou XH, Liu J (2014). Conserved miRNAs and their response to salt stress in wild eggplant Solanum linnaeanum roots. Int J Mol Sci.

[12] Ding D, Zhang L, Wang H, Liu Z, Zhang Z, Zheng Y (2009). Differential expression of miRNAs in response to salt stress in maize roots. Ann Bot.

[13] Sun X, Xu L, Wang Y, Yu R, Zhu X, Luo X, Gong Y, Wang R, Limera C, Zhang K (2015). Identification of novel and salt-responsive miRNAs to explore miRNA-mediated regulatory network of salt stress response in radish (*Raphanus sativus* L.). BMC Genomics.

[14] Goswami K, Tripathi A, Sanan-Mishra N: Comparative miRomics of Salt-Tolerant and Salt-Sensitive Rice. *Journal of integrative bioinformatics* 2017, 14(1). 10.1515/jib-2017-0002 PMC6042804 28637931

[15] Carnavale Bottino M, Rosario S, Grativol C, Thiebaut F, Rojas CA, Farrineli L, Hemerly AS, Ferreira PC (2013). High-throughput sequencing of small RNA transcriptome reveals salt stress regulated microRNAs in sugarcane. PLoS One.

[16] Zhang Q, Zhao C, Li M, Sun W, Liu Y, Xia H, Sun M, Li A, Li C, Zhao S (2013). Genome-wide identification of *Thellungiella salsuginea* microRNAs with putative roles in the salt stress response. BMC Plant Biol.

[17] Bai Q, Wang X, Chen X, Shi G, Liu Z, Guo C, Xiao K (2018). Wheat miRNA TaemiR408 acts as an essential mediator in plant tolerance to pi deprivation and salt stress via modulating stress-associated physiological processes. Front Plant Sci.

[18] Yang X, Wang L, Yuan D, Lindsey K, Zhang X (2013). Small RNA and degradome sequencing reveal complex miRNA regulation during cotton somatic embryogenesis. J Exp Bot.

[19] Tian Y, Tian Y, Luo X, Zhou T, Huang Z, Liu Y, Qiu Y, Hou B, Sun D, Deng H (2014). Identification and characterization of microRNAs related to salt stress in broccoli, using high-throughput sequencing and bioinformatics analysis. BMC Plant Biol.

[20] Fu R, Zhang M, Zhao Y, He X, Ding C, Wang S, Feng Y, Song X, Li P, Wang B: Identification of Salt Tolerance-related microRNAs and Their Targets in Maize (*Zea mays* L.) Using High-throughput Sequencing and Degradome Analysis. Front Plant Sci 2017, 8:864. 10.3389/fpls.2017.00864 PMC5445174 28603532

[21] Liu B (2017). Sun G: microRNAs contribute to enhanced salt adaptation of the autopolyploid Hordeum bulbosum compared with its diploid ancestor. The Plant journal : for cell and molecular biology.

[22] Zhou M, Li D, Li Z, Hu Q, Yang C, Zhu L, Luo H (2013). Constitutive expression of a miR319 gene alters plant development and enhances salt and drought tolerance in transgenic creeping bentgrass. Plant Physiol.

[23] Chen Z, Hu L, Han N, Hu J, Yang Y, Xiang T, Zhang X, Wang L (2015). Overexpression of a miR393-resistant form of transport inhibitor response protein 1 (mTIR1) enhances salt tolerance by increased osmoregulation and Na+ exclusion in *Arabidopsis thaliana*. Plant & cell physiology.

[24] Song JB, Gao S, Sun D, Li H, Shu XX (2013). Yang ZM: miR394 and LCR are involved in *Arabidopsis* salt and drought stress responses in an abscisic acid-dependent manner. BMC Plant Biol.

[25] Kim JY, Lee HJ, Jung HJ, Maruyama K, Suzuki N, Kang H (2010). Overexpression of microRNA395c or 395e affects differently the seed germination of *Arabidopsis thaliana* under stress conditions. Planta.

[26] Chen L, Luan Y, Zhai J (2015). Sp-miR396a-5p acts as a stress-responsive genes regulator by conferring tolerance to abiotic stresses and susceptibility to Phytophthora nicotianae infection in transgenic tobacco. Plant Cell Rep.

[27] Wang W, Liu D, Chen D, Cheng Y, Zhang X, Song L, Hu M, Dong J, Shen F (2019). MicroRNA414c affects salt tolerance of cotton by regulating reactive oxygen species metabolism under salinity stress. RNA Biol.

[28] Yuan S, Li Z, Li D, Yuan N, Hu Q, Luo H (2015). Constitutive expression of Rice MicroRNA528 alters plant development and enhances tolerance to salinity stress and nitrogen starvation in creeping Bentgrass. Plant Physiol.

[29] Tuteja N (2007). Mechanisms of high salinity tolerance in plants. Methods Enzymol.

[30] Roy SJ, Negrao S, Tester M (2014). Salt resistant crop plants. Curr Opin Biotechnol.

[31] Marakli S (2018). Identification and functional analyses of new sesame miRNAs (*Sesamum indicum* L.) and their targets. Mol Biol Rep.

[32] Xie F, Wang Q, Sun R, Zhang B (2015). Deep sequencing reveals important roles of microRNAs in response to drought and salinity stress in cotton. J Exp Bot.

[33] Yin Z, Li Y, Yu J, Liu Y, Li C, Han X, Shen F (2012). Difference in miRNA expression profiles between two cotton cultivars with distinct salt sensitivity. Mol Biol Rep.

[34] Liu HH, Tian X, Li YJ, Wu CA, Zheng CC (2008). Microarray-based analysis of stress-regulated microRNAs in *Arabidopsis thaliana*. Rna.

[35] Yoon S, De Micheli G: Prediction of regulatory modules comprising microRNAs and target genes. *Bioinformatics* 2005, 21 Suppl 2:ii93–100. 10.1093/bioinformatics/bti1116 16204133

[36] Lai X, Bhattacharya A, Schmitz U, Kunz M, Vera J, Wolkenhauer O (2013). A systems' biology approach to study microRNA-mediated gene regulatory networks. Biomed Res Int.

[37] Abdul Hadi LH, Xuan Lin QX, Minh TT, Loh M, Ng HK, Salim A, Soong R (2018). Benoukraf T: miREM: an expectation-maximization approach for prioritizing miRNAs associated with gene-set. BMC bioinformatics.

[38] Li XL, Yang X, Hu YX, Yu XD, Li QL (2014). A novel NAC transcription factor from Suaeda liaotungensis K. enhanced transgenic *Arabidopsis* drought, salt, and cold stress tolerance. Plant Cell Rep.

[39] Yu X, Liu Y, Wang S, Tao Y, Wang Z, Shu Y, Peng H, Mijiti A, Wang Z, Zhang H (2016). CarNAC4, a NAC-type chickpea transcription factor conferring enhanced drought and salt stress tolerances in *Arabidopsis*. Plant Cell Rep.

[40] Wang L, Li Z, Lu M, Wang Y: ThNAC13, a NAC transcription factor from Tamarix hispida, Confers Salt and Osmotic Stress Tolerance to Transgenic Tamarix and *Arabidopsis Frontiers in plant science* 2017, 8:635. 10.3389/fpls.2017.00635 PMC5405116 28491072

[41] Ju YL, Yue XF, Min Z, Wang XH, Fang YL, Zhang JX (2020). VvNAC17, a novel stress-responsive grapevine (Vitis vinifera L.) NAC transcription factor, increases sensitivity to abscisic acid and enhances salinity, freezing, and drought tolerance in transgenic *Arabidopsis*. Plant physiology and biochemistry : PPB.

[42] Guo Y, Halfter U, Ishitani M, Zhu JK (2001). Molecular characterization of functional domains in the protein kinase SOS2 that is required for plant salt tolerance. Plant Cell.

[43] Flowers TJ (2004). Improving crop salt tolerance. J Exp Bot.

[44] Katiyar-Agarwal S, Zhu J, Kim K, Agarwal M, Fu X, Huang A, Zhu JK (2006). The plasma membrane Na+/H+ antiporter SOS1 interacts with RCD1 and functions in oxidative stress tolerance in *Arabidopsis*. Proc Natl Acad Sci U S A.

[45] Barrero JM, Rodriguez PL, Quesada V, Piqueras P, Ponce MR, Micol JL (2006). Both abscisic acid (ABA)-dependent and ABA-independent pathways govern the induction of NCED3, AAO3 and ABA1 in response to salt stress. Plant Cell Environ.

[46] Munns R, Tester M (2008). Mechanisms of salinity tolerance. Annu Rev Plant Biol.

[47] Hashem A, Abd Allah EF, Alqarawi AA, Al-Huqail AA, Shah MA: Induction of Osmoregulation and Modulation of Salt Stress in *Acacia gerrardii* Benth. by Arbuscular Mycorrhizal Fungi and Bacillus subtilis (BERA 71). Biomed Res Int 2016, 2016:6294098. 10.1155/2016/6294098 PMC5002495 27597969

[48] Zhang M, Smith JA, Harberd NP, Jiang C (2016). The regulatory roles of ethylene and reactive oxygen species (ROS) in plant salt stress responses. Plant Mol Biol.

[49] Zhu JK (2016). Abiotic stress signaling and responses in plants. Cell.

[50] Eldem V, Celikkol Akcay U, Ozhuner E, Bakir Y, Uranbey S, Unver T (2012). Genome-wide identification of miRNAs responsive to drought in peach (*Prunus persica*) by high-throughput deep sequencing. PLoS One.

[51] Ren Y, Chen L, Zhang Y, Kang X, Zhang Z, Wang Y (2013). Identification and characterization of salt-responsive microRNAs in Populus tomentosa by high-throughput sequencing. Biochimie.

[52] Yang T, Wang Y, Teotia S, Wang Z, Shi C, Sun H, Gu Y, Zhang Z, Tang G (2019). The interaction between miR160 and miR165/166 in the control of leaf development and drought tolerance in *Arabidopsis*. Sci Rep.

[53] Zhang Y, Li D, Zhou R, Wang X, Dossa K, Wang L, Zhang Y, Yu J, Gong H, Zhang X (2019). Transcriptome and metabolome analyses of two contrasting sesame genotypes reveal the crucial biological pathways involved in rapid adaptive response to salt stress. BMC Plant Biol.

[54] Han H, Wang Q, Wei L, Liang Y, Dai J, Xia G, Liu S (2018). Small RNA and degradome sequencing used to elucidate the basis of tolerance to salinity and alkalinity in wheat. BMC Plant Biol.

[55] Tian Y, Shang Y, Guo R, Chang Y, Jiang Y (2019). Salinity stress-induced differentially expressed miRNAs and target genes in sea cucumbers *Apostichopus japonicus*. Cell Stress Chaperones.

[56] Friedlander MR, Mackowiak SD, Li N, Chen W (2012). Rajewsky N: miRDeep2 accurately identifies known and hundreds of novel microRNA genes in seven animal clades. Nucleic Acids Res.

[57] Wen M, Shen Y, Shi S (2012). Tang T: miREvo: an integrative microRNA evolutionary analysis platform for next-generation sequencing experiments. BMC bioinformatics.

[58] Li B, Qin Y, Duan H, Yin W, Xia X (2011). Genome-wide characterization of new and drought stress responsive microRNAs in *Populus euphratica*. J Exp Bot.

[59] Ma X, Zhang X, Zhao K, Li F, Li K, Ning L, He J, Xin Z, Yin D: Small RNA and Degradome Deep Sequencing Reveals the Roles of microRNAs in Seed Expansion in Peanut (*Arachis hypogaea* L.). Front Plant Sci 2018, 9:349. 10.3389/fpls.2018.00349 PMC5890158 29662498

[60] Jian H, Ma J, Wei L, Liu P, Zhang A, Yang B, Li J, Xu X, Liu L: Integrated mRNA, sRNA, and degradome sequencing reveal oilseed rape complex responses to *Sclerotinia sclerotiorum* (Lib.) infection. *Scientific reports* 2018, 8(1):10987. 10.1038/s41598-018-29365-y PMC6054686 30030454

[61] Zhu H, Zhang Y, Tang R, Qu H, Duan X, Jiang Y (2019). Banana sRNAome and degradome identify microRNAs functioning in differential responses to temperature stress. BMC Genomics.

[62] Young MD, Wakefield MJ, Smyth GK, Oshlack A (2010). Gene ontology analysis for RNA-seq: accounting for selection bias. Genome Biol.

[63] Livak KJ, Schmittgen TD (2001). Analysis of relative gene expression data using real-time quantitative PCR and the 2^−∆∆Ct^ method. Methods.

