# Supplementary figures and images for: Integrated small RNA and Degradome sequencing provide insights into salt tolerance in sesame (Sesamum indicum L.)
Source: BMC Genomics. 2020 Jul 18;21:494. doi: 10.1186/s12864-020-06913-3 (PMC7368703; doi:10.1186/s12864-020-06913-3)

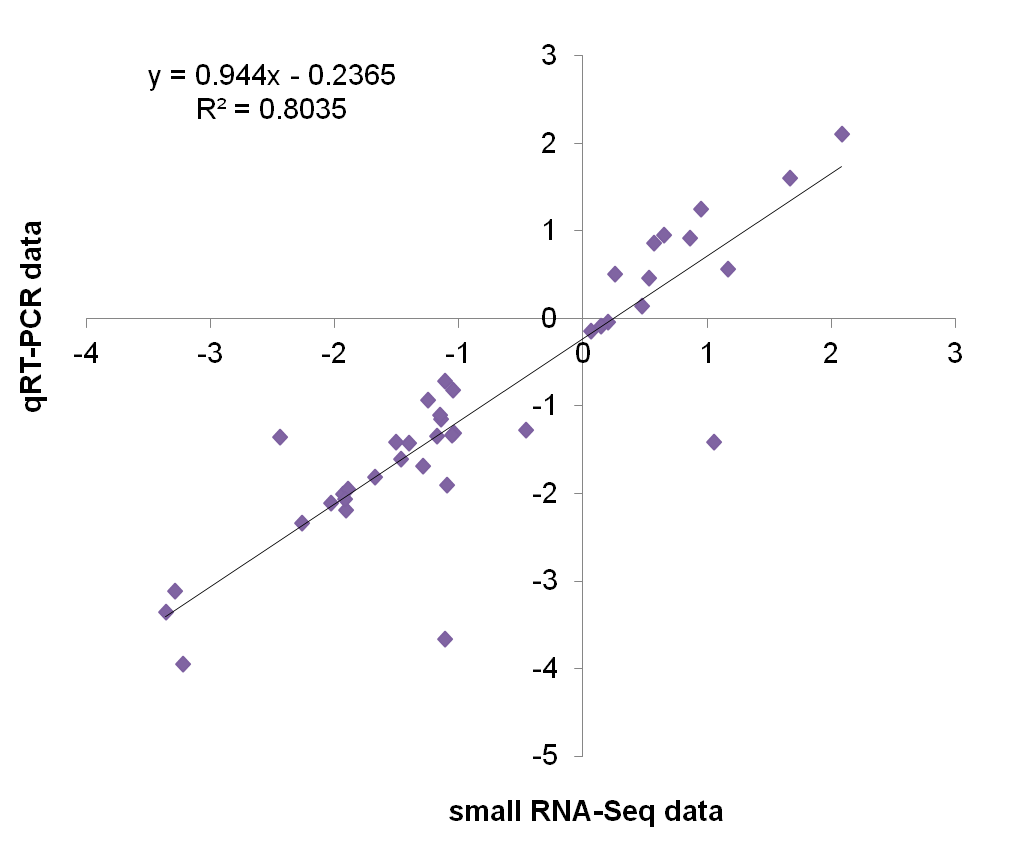

Supplement: Supplementary file 2 — Additional file 2 Fig. S1. Correlation analysis between qRT-PCR and small RNA sequencing data based on log2fold change of 10 selected miRNAs. [file 12864_2020_6913_MOESM2_ESM.tif]
